# The association of sugar-sweetened beverage intake and conicity index with mental health symptoms among Chinese adolescents

**DOI:** 10.3389/fpsyg.2026.1929362

**Published:** 2026-10-02

**Authors:** Linlin Li, Yuqiang Li, Huijun Yan, Hui Wang, Guangtao Zhao, Min Zhang

**Affiliations:** 1 School of Physical Education, Huanghuai University, Zhumadian, China; 2 College of Physical Education and Health, East China Normal University, Shanghai, China; 3 College of Physical Education and Health Management, College of Physical Education, Henan Finance University, Zhengzhou, China; 4 College of Sports Science, Hefei Normal University, Hefei, Anhui, China; 5 School of Physical Education, Chizhou University, Chizhou, China

**Keywords:** adolescents, Chinese, conicity index, mental health symptoms, sugar-sweetened beverage intake

## Abstract

**Background:**

Mental health issues among adolescents are becoming increasingly prominent, and sugar-sweetened beverage (SSB) intake and the conicity index (CI) may be associated with these issues. However, current research provides limited evidence on the combined effects of these two factors. This study aims to investigate the association between SSB intake and CI and mental health symptoms among Chinese adolescents.

**Methods:**

Using a cross-sectional design, this study assessed 42,382 middle school students (21,156 males and 21,226 females) nationwide in China in 2024. A questionnaire was used to collect data on the frequency of SSB consumption, mental health symptoms (emotional problems, behavioral problems, and social adjustment difficulties), and covariates. CI was calculated using waist circumference, height, and weight. Logistic regression and joint effects analysis were employed, with stepwise adjustment for age, mode of commuting, breakfast frequency, and moderate-to-vigorous physical activity.

**Results:**

Among 42,382 adolescents, the prevalence of mental health symptoms was 20.9% (males 21.4% vs. females 20.5%). SSB intake ≥5 times/week (OR = 1.66) and the highest CI quartile (OR = 1.17) were independently associated with mental health symptoms. The combined effect was strongest for high SSB with highest CI (OR = 2.12), with a stronger combined association among individuals with both exposures, particularly in females (OR = 3.41).

**Conclusion:**

SSB intake and CI were both independently associated with for mental health symptoms among Chinese adolescents, and there is a synergistic effect between the two. Findings suggest that lower SSB intake and lower central adiposity are associated with adolescents’ mental health; gender differences suggest the need to develop targeted intervention strategies.

## 1 Introduction

Mental health issues have become one of the most serious public health challenges facing adolescents worldwide (Duncan and Sheldrick, 2025). According to data from the World Health Organization, approximately 10 to 20% of adolescents worldwide have experienced mental health disorders to varying degrees, with about 50% of these disorders emerging before the age of 14, posing a serious threat to their health during adolescence and into adulthood. In 2021, the number of adolescents with depression worldwide reached 57.49 million, an increase of 49.41% from 1990—a rise of nearly half (Sun et al., 2026). Mental health symptoms primarily manifest as emotional problems (such as depression, tension, and anxiety), behavioral problems (such as social withdrawal and aggressive behavior), and difficulties in social adaptation. If these symptoms persist, they can develop into serious mental disorders, having far-reaching negative impacts on adolescents’ academic performance, interpersonal relationships, and lifelong development. In recent years, the prevalence of mental health symptoms among adolescents worldwide has continued to rise, becoming one of the issues of great concern to scholars and public health policymakers in various countries. The mental health situation among adolescents in China is particularly difficult and is rapidly worsening. A systematic review and meta-analysis covering mainland China showed that the overall prevalence of depressive symptoms among middle school students is approximately 26.17% (Zhou et al., 2024). Data from a nationwide survey indicate that the overall prevalence of mental health symptoms among adolescents is approximately 20.9%, with boys (21.4%) showing a slightly higher rate than girls (20.5%) (Wang R. et al., 2025). Mental health issues have become the second-largest source of disease burden in China today, second only to cardiovascular disease. Among adolescents, mental disorders such as anxiety and depression are affecting younger and younger age groups and show a continuing upward trend (Yang et al., 2024). Against this backdrop, systematically identifying modifiable risk factors for mental health symptoms in adolescents and implementing targeted preventive interventions has become a critical issue requiring urgent breakthroughs in China’s public health sector.

The consumption of sugar-sweetened beverages (SSB) has been one of the most prominent changes in adolescents’ lifestyles in recent years. SSB are defined as beverages containing free sugars, including soft drinks, certain fruit juices, energy drinks, and caffeinated beverages. Adolescents are the group with the highest consumption of SSB, which have become the primary source of free sugars in their diets, posing serious negative impacts on their physical and mental health (Mayer-Davis et al., 2020). A demographic study covering 185 countries shows that global SSB consumption among adolescents increased by 23% between 1990 and 2018, rising by 0.68 servings per week (Sun et al., 2026). A large body of epidemiological evidence indicates a significant association between SSB consumption and mental health in adolescents. A systematic review and meta-analysis published by Khaled et al. in 2026 included 9 studies—7 cross-sectional and 2 longitudinal. The results showed that high SSB intake was significantly associated with a 34% increased risk of anxiety disorders among adolescents (OR = 1.34); the longitudinal studies also found that this association persisted during follow-up periods of more than one year (Khaled et al., 2026). A longitudinal study of 13,887 Canadian adolescents (aged 14–18) found that baseline SSB intake was significantly associated with more severe depressive symptoms (*β* = 0.04), anxiety symptoms (*β* = 0.02), and lower psychological well-being (*β* = −0.03) at the 1-year follow-up (Dabravolskaj et al., 2024). Studies of Chinese adolescents have similarly confirmed this association. A multicenter cross-sectional survey involving 42,832 adolescents aged 12–17 found that adolescents who consumed SSBs ≥4 times per week had a significantly increased risk of mental health symptoms, and that SSB consumption and muscle strength had a combined effect on mental health symptoms (Zhou et al., 2025). A systematic review analyzed 57 studies examining the association between SSB consumption and adolescents’ sleep and mental health; the results support the possibility of a bidirectional association between SSB intake and adverse mental health outcomes (Sun et al., 2026). This evidence suggests that SSB consumption is not only a risk factor for metabolic diseases but also a modifiable risk factor for adolescent mental health that cannot be overlooked.

The Conicity Index (CI) is an important anthropometric measure for assessing body fat distribution, particularly central obesity. Unlike the Body Mass Index (BMI), which relies solely on weight, the CI incorporates waist circumference, height, and weight to more accurately reflect the degree of visceral fat accumulation and the characteristics of body fat distribution (Wang et al., 2023). Previous studies have shown that CI is closely associated with various metabolic disorders, including adolescent metabolic syndrome, dyslipidemia, and hepatic steatosis (Hao et al., 2025). However, existing research on CI has largely focused on its association with physical diseases, and studies examining the relationship between CI and mental health are extremely limited. Although some studies suggest that new obesity indicators, including CI, “may be more accurate in identifying individuals with depression,” there remains a lack of empirical research directly investigating the association between CI and mental health symptoms in adolescents. The few relevant studies mostly use single indicators such as waist circumference (WC). A study of Chinese adolescents found that waist circumference was negatively correlated with mental health and partially mediated the relationship between screen time and mental health; that is, excessive screen time may indirectly harm mental health by increasing waist circumference (De Moraes et al., 2023). In recent years, although some studies have begun to examine the impact of combined exposure to SSB intake and other body fat indicators—such as the weight-adjusted waist circumference index (WWI) and the body roundness index (BRI)—on mental health symptoms, the combined effect of CI—a sensitive indicator of central obesity—and SSB intake on mental health symptoms has not yet been fully explored. Among adolescents in China, studies examining the independent and combined effects of CI and mental health symptoms are particularly rare.

Based on the current state of research and the current situation regarding mental health symptoms among adolescents, this study utilizes data from a large-scale cross-sectional survey of Chinese adolescents (*n* = 42,382) to analyze the distribution characteristics of SSB intake, conicity index (CI), and mental health symptoms among Chinese adolescents, and to explore the independent associations between SSB intake, CI, and mental health symptoms. Building on this, the study analyzes the combined effects of SSB intake and CI on mental health symptoms. The aim is to provide a scientific basis for targeted prevention and personalized interventions for the mental health of Chinese adolescents.

## 2 Methods

### 2.1 Participants

This cross-sectional survey and evaluation employed a multistage, stratified, cluster-random sampling method and was conducted between March and November 2024. First, the country was divided into five sampling strata based on China’s five major geographic regions (Eastern, Central, Western, Northeastern, and Southwestern). Taking into account each province’s level of economic development and the ratio of urban to rural population, probability sampling proportional to population size was used to select 2–3 provinces from each geographic region, resulting in a total of 12 provinces chosen as first-level sampling units. Next, within each selected province, the population was stratified by urban and rural areas. Using cluster sampling, 3–4 secondary schools (including junior high and senior high schools) were randomly selected from both urban and rural areas, resulting in approximately 160 schools as second-level sampling units. Finally, for each of the six grade levels (7th through 12th) in the sampled middle and high schools, a simple random sample of 1–2 classes per grade was selected, and a survey was administered to all students in those classes. The inclusion criteria for participants in this study were: (1) currently enrolled middle and high school students aged 12–17; (2) willing to participate and able to complete the questionnaire independently. The exclusion criteria for participants were: (1) those with severe chronic physical illnesses, such as congenital heart disease, severe liver or kidney disease, or a self-reported prior diagnosis of a mental disorder made by a qualified psychiatrist (information collected via the parental consent form); (2) those with missing data on key covariates, such as physical activity or breakfast frequency; (3) those who did not sign the informed consent form or refused to cooperate with the physical examination. Ultimately, 42,382 school-aged adolescents aged 12–17 were included as the valid analysis sample. All participants in this study were minors, and written informed consent was obtained from the legal guardians of all participating minors prior to the start of the study; participating students additionally gave oral assent at the time of the survey. The study was approved by the Human Ethics Committee of Chizhou University (CZXY-2026-TYXY-006).

### 2.2 Sugar-sweetened beverage intake (SSB)

The frequency of SSB consumption was assessed using a self-administered questionnaire based on the SSB consumption assessment module of the internationally recognized Health Behaviors in School-aged Children (HBSC) survey (Lazzeri et al., 2013). This questionnaire has been extensively validated and applied among adolescent populations in many countries worldwide (Liu et al., 2010). Participants were asked to recall how many times they consumed various types of SSB over the past week. SSBs are defined as liquid beverages containing added sugars, such as sucrose, fructose, glucose, and corn syrup, and specifically include: (1) carbonated beverages (e.g., Coke, Sprite, etc.); (2) fruit and vegetable juice beverages (other than 100% pure fruit juice); (3) sugar-sweetened tea beverages (e.g., iced black tea, milk tea, etc.); (4) energy drinks and sports drinks; (5) sugar-sweetened flavored dairy beverages; (6) other SSB (e.g., sour plum soup, mung bean paste, etc.). This definition excludes 100% pure fruit juice, sugar-free beverages, and unsweetened beverages prepared at home.

The SSB frequency options were set as follows: never consumed, ≤2 times per week, 3–4 times per week, 5–6 times per week, and ≥7 times per week. Based on previous literature and the actual distribution of SSB consumption among Chinese adolescents, this study categorized SSB intake frequency into three levels: ≤2 times per week (low frequency), 3–4 times per week (moderate frequency), and ≥5 times per week (high frequency) (Tasevska et al., 2017). The questionnaire was validated through a pilot study before the formal survey, with a test–retest reliability coefficient (Pearson correlation coefficient) of 0.82. To ensure the questionnaire’s cultural appropriateness, the original HBSC version underwent back-translation and expert review, retaining its core question structure and scoring criteria. The Chinese version was reviewed and validated for content validity by five experts in public health and adolescent health.

### 2.3 Conicity index (CI)

The CI is an important anthropometric indicator for assessing body fat distribution, particularly central obesity. Unlike the Body Mass Index (BMI), which relies solely on weight, the CI incorporates waist circumference, height, and weight to more accurately reflect the degree of visceral fat accumulation and the characteristics of body fat distribution. The CI was first proposed by Mueller et al. (1996), and its formula is as follows:

$$\begin{array}{l} \mathrm{CI}=\text{waist circumference}\;(m)\\ /[0.109\times \surd (\text{body weight}\;(\mathrm{kg})/\text{height}\;(m))] \end{array}$$

Mueller et al. (1996); note: this is the conicity-index paper, distinct from Mueller et al. (1996), which concerns salivary-gland function].

In this context, 0.109 is a constant calculated based on the assumption of human body density.

In this study, staff measured the waist circumference, height, and weight of all participants in accordance with standardized operating procedures. Waist circumference was measured at the end of exhalation, 1 centimeter above the umbilicus, with a reading taken horizontally around the abdomen, accurate to 0.1 centimeter; height was measured using a stadiometer, accurate to 0.1 centimeter; and weight was measured using an electronic scale, accurate to 0.1 kilogram. After the measurements were completed, the CI value for each adolescent was calculated individually using the Valdez formula. To further analyze the dose–response relationship between CI and mental health symptoms, this study included CI values stratified by age and sex and grouped into quartiles (Quartiles 1–4) for statistical analysis. As a sensitive indicator of abdominal fat distribution, CI has been widely used and validated in adolescent populations and can effectively identify central obesity and its associated health risks.

### 2.4 Mental health symptoms

This study used the Brief Instrument on Psychological Health of Youths (BIOPHY), developed by Shuman et al. (2020) to assess mental health symptoms among Chinese adolescents (Zhang et al., 2023). The BIOPHY questionnaire consists of 15 items, divided into three dimensions: emotional problems (items 1–7), behavioral problems (items 8, 10, 11, 13), and social adjustment difficulties (items 9, 12, 14, 15). Each item has six response options corresponding to different durations ranging from “lasting more than 3 months” to “none or lasting less than 1 week.” Scoring uses a binary classification system: options 1, 2, and 3 (i.e., duration exceeding 1 month) are scored as 1 point, while options 4, 5, and 6 are scored as 0 points. The total score is calculated by summing the scores across all 15 items, with each item ranging from 0 to 15 points; corresponding dimension scores can also be calculated for each dimension. Cutoff criteria were established based on the 90th percentile (P90) of national data: a total score of 7 or higher indicates the presence of mental health problems. The cutoff values for the three dimensions are as follows: emotional problems ≥ 4, behavioral problems ≥ 1, and social adjustment difficulties ≥ 2. This questionnaire assesses the persistence of participants’ mental health symptoms over the past 3 months. Regarding reliability and validity testing, exploratory factor analysis showed a cumulative variance explained of 57.39%; the Cronbach’s α for the total questionnaire was 0.928, and the split-half reliability was 0.909, both of which meet psychological assessment standards (Zheng et al., 2025).

### 2.5 Covariates

The covariates included in this study were age, gender, commuting to and from school, breakfast frequency, moderate-to-vigorous physical activity, sleep duration on a typical weekday, parental education level (used as a proxy for family socioeconomic status), self-reported academic pressure, smoking (ever tried), and alcohol use (ever tried).

Commuting to and from school was assessed using a self-reported questionnaire. Commuting to and from school was categorized into active and passive methods (Bere and Bjorkelund, 2009). Active methods include walking or biking to and from school; passive methods include traveling by private car or public transportation. This method draws on the “Mode and Frequency of Commuting To and From School Questionnaire” used in the Pediatric Active Commuting (PACO) study, which has been shown to have good feasibility and reliability, with Kappa coefficients ranging from 0.61 to 0.94 (Bere and Bjorkelund, 2009). Typically, a single question is asked: “How do you usually get to and from school?” The options include walking, biking, private car, public transportation, and so on.

Breakfast frequency was assessed using a self-report questionnaire that asked how many days participants ate breakfast in the past week. This assessment method was based on the standardized question used in the Global School-based Health Survey (GSHS): “How often do you usually eat breakfast (more than a glass of milk or juice)?”(Segura-Diaz et al., 2020). The World Health Organization’s standard procedure for school health surveys was followed, allowing participants to select the number of days per week they ate breakfast based on their actual habits. In this study, breakfast frequency was categorized into three groups: ≥5 times/week, 3–4 times/week, and ≤2 times/week.

Moderate-to-Vigorous Physical Activity (MVPA) was assessed using the International Physical Activity Questionnaire Short Form (IPAQ-SF) (Lee et al., 2011). The IPAQ is one of the most widely used self-report tools for physical activity worldwide. It has proven effective for assessing physical activity among Chinese adolescents (Wang et al., 2013). The questionnaire asked participants about the number of days in the past 7 days they engaged in walking, moderate-intensity physical activity (such as brisk walking or doubles tennis), and vigorous-intensity physical activity (such as jogging, brisk cycling, or singles tennis), as well as the duration of each activity per day. MVPA duration was calculated as the sum of moderate- and vigorous-intensity physical activity durations. In this study, MVPA was categorized into three groups: <30 min/day, 30–60 min/day, and >60 min/day.

### 2.6 Statistical analysis

All statistical analyses were performed using SPSS software, with two-sided tests and a significance level of α = 0.05. Continuous variables (age, height, weight, waist circumference, and CI) were described as mean ± standard deviation (Mean ± SD), and comparisons between groups were analyzed using the independent samples t-test; categorical variables (gender, mode of commuting to school, breakfast frequency, MVPA, SSB intake, CI quartile groups, and each dimension of mental health symptoms) were expressed as frequencies (percentages), and comparisons between groups were analyzed using the χ^2^ test.

First, we used descriptive analysis to compare differences in the distributions of various variables between genders and in covariates between the group with mental health symptoms and the group without mental health symptoms. Second, we used univariate chi-square tests to analyze the associations between SSB intake and CI quartile groups, on the one hand, and mental health symptoms and their various dimensions, on the other.

Next, in the multivariate analysis, we constructed binary logistic regression models with the presence of mental health problems (yes/no, defined as a total score≥7) as the dependent variable. We established three stepwise adjustment models: Model 1 was the crude (unadjusted) model; Model 2 was adjusted for age based on Model 1; and Model 3 was further adjusted for mode of commuting, breakfast frequency, and MVPA based on Model 2. Using SSB intake ≤ 2 times per week and the lowest quartile (Q1) of CI as reference groups, we calculated the odds ratios (OR) and their 95% confidence intervals (CI) for each group.

Finally, to examine the combined effect of SSB intake and CI, 12 interaction combinations were constructed based on SSB intake (≤2 times/week, 3–4 times/week, ≥5 times/week) and CI quartiles (Q1–Q4). Using “SSB ≤ 2 times/week and CI Q1” as the common reference group, we performed both binary logistic regression (presence vs. absence of mental health symptoms, defined as a total BIOPHY score ≥7) and, as a sensitivity analysis, ordered logistic regression treating the total BIOPHY score (0–15) as an ordinal outcome to estimate the OR values and 95% CIs for each interaction group; the proportional-odds assumption was assessed using the Brant test and was not violated (*p* > 0.05). All models were adjusted for age, gender (in the total-sample model), mode of commuting to school, breakfast frequency, MVPA, sleep duration, parental education, academic pressure, smoking, and alcohol use. Subgroup analyses were also conducted by gender to assess potential effect moderators. To formally test whether the observed joint associations reflect statistical interaction, we additionally estimated the relative excess risk due to interaction (RERI) and the multiplicative interaction term (SSB × CI) for each SSB-CI combination. All regression models underwent collinearity diagnostics; a variance inflation factor (VIF) < 5 indicated no significant collinearity.

## 3 Results

Table 1 presents a comparison of baseline data, stratified by gender, for 42,382 Chinese adolescents, comprising 21,156 boys and 21,226 girls. Among continuous variables, the mean values for age, height, weight, waist circumference, and CI were all significantly higher in boys than in girls (all *p <* 0.05). Regarding categorical variables, the proportion of boys who engaged in active commuting (46.9%) was higher than that of girls (40.6%); girls had a higher proportion of passive commuting. There was no significant difference in breakfast frequency between boys and girls (*p* = 0.171). Regarding moderate-to-vigorous physical activity (MVPA), 18.9% of boys engaged in >60 min per day, which was significantly higher than the 9.2% among girls; 53.8% of girls engaged in <30 min per day. Frequent consumption of SSB (≥5 times/week) was significantly higher among male students (17.3%) than among female students (11.7%). When stratified by quartiles, 32.1% of male students were in the highest quartile (Q4), compared to only 17.8% of female students. Regarding mental health symptoms, there was no difference in the prevalence of emotional problems between boys and girls (*p* = 0.709); however, the prevalence of behavioral problems, social adjustment difficulties, and overall mental health symptoms was significantly higher among boys than among girls. The prevalence of overall mental health symptoms was 21.4% for boys and 20.5% for girls (*p* = 0.034).

**Table 1:** A gender comparison of the basic characteristics of Chinese youth.

Classification	Male	Female	Total	*χ*^2^ /*t*-value	*p*-value
Number	21,156	21,226	42,382		
Age (years)	14.63 ± 1.62	14.70 ± 1.65	14.67 ± 1.64	4.249	0.027
Height	169.23 ± 9.29	160.93 ± 6.48	165.07 ± 9.02	106.703	<0.001
Weight	58.84 ± 13.44	51.17 ± 9.23	55.00 ± 12.15	68.538	<0.001
Waist circumference	71.70 ± 11.44	66.15 ± 9.16	68.92 ± 10.73	55.062	<0.001
Conicity index	1.12 ± 0.14	1.08 ± 0.13	1.10 ± 0.13	32.389	<0.001
Commuting to and from school				171.022	<0.001
Active methods	9,928(46.9)	8,623(40.6)	18,551(43.8)		
Passive methods	11,228(53.1)	12,603(59.4)	23,831(56.2)		
Frequency of breakfast				3.535	0.171
≥5 times/week	17,576(83.1)	17,751(83.6)	35,327(83.4)		
3–4 times/week	2,675(12.6)	2,636(12.4)	5,311(12.5)		
≤2 times/week	905(4.3)	839(4.0)	1744(4.1)		
Duration of moderate-to-vigorous physical activity (MVPA)				1397.539	<0.001
<30 min/day	8,016(37.9)	11,417(53.8)	19,433(45.9)		
30–60 min/day	9,144(43.2)	7,861(37.0)	17,005(40.1)		
>60 min/day	3,996(18.9)	1948(9.2)	5,944(14.0)		
Sugar-sweetened beverage intake				476.294	<0.001
≤2 times/week	6,166(29.1)	7,978(37.6)	14,144(33.4)		
3–4 times/week	11,328(53.5)	10,775(50.8)	22,103(52.2)		
≥5 times/week	3,662(17.3)	2,473(11.7)	6,135(14.5)		
Quartile of conicity index				1553.149	<0.001
Quartile 1	4,186(19.8)	6,422(30.3)	10,608(25.0)		
Quartile 2	4,563(21.6)	5,986(28.2)	10,549(24.9)		
Quartile 3	5,612(26.5)	5,037(23.7)	10,649(25.1)		
Quartile 4	6,795(32.1)	3,781(17.8)	10,576(25.0)		
Emotional problems	5,880(27.8)	5,865(27.6)	11,745(27.7)	0.139	0.709
Behavioral problems	5,944(28.1)	5,393(25.4)	11,337(26.7)	39.086	<0.001
Social adjustment difficulties	3,883(18.4)	3,536(16.7)	7,419(17.5)	21.088	<0.001
Mental health symptoms	4,520(21.4)	4,357(20.5)	8,877(20.9)	4.498	0.034

Table 2 presents univariate comparisons between two groups of adolescents with and without mental health symptoms. The total sample consisted of 42,382 participants, including 33,505 without symptoms and 8,877 with symptoms (20.9%). Except for age, where differences between groups were not statistically significant (*p* = 0.070), significant differences were observed between groups for all other indicators (*p <* 0.05). Adolescents with mental health symptoms had higher mean values for height, weight, waist circumference, and CI; the prevalence of mental health symptoms was higher among boys than among girls. The prevalence of mental health symptoms was significantly higher among those with passive commuting, low breakfast frequency, less than 30 min of daily MVPA, high-frequency consumption of SSB, and those in the upper quartile of the CI, and this relationship exhibited a gradient pattern. The lower the breakfast frequency, the shorter the duration of physical activity, the more frequent the consumption of SSB, and the higher the CI quartile, the higher the prevalence of mental health symptoms.

**Table 2:** Univariate comparison of the presence of mental health symptoms among Chinese adolescents.

Classification	**Mental health symptoms**		***χ*** ^**2**^ **/*t*-value**	***P*-value**
	**No**	**Yes**		
Number	33,505	8,877		
Age (years)	14.66 ± 1.65	14.70 ± 1.58	1.814	0.070
Height	164.97 ± 9.02	165.48 ± 9.00	4.775	<0.001
Weight	54.71 ± 11.96	56.09 ± 12.77	9.512	<0.001
Waist circumference	68.65 ± 10.42	69.94 ± 11.77	10.086	<0.001
Conicity index	1.10 ± 0.13	1.11 ± 0.14	5.158	<0.001
Sex			4.498	0.034
Male	16,636(78.6)	4,520(21.4)		
Female	16,869(79.5)	4,357(20.5)		
Commuting to and from school			5.970	0.015
Active methods	14,767(79.6)	3,784(20.4)		
Passive methods	18,738(78.6)	5,093(21.4)		
Frequency of breakfast			413.106	<0.001
≥5 times/week	28,475(80.6)	6,852(19.4)		
3–4 times/week	3,938(74.1)	1,373(25.9)		
≤2 times/week	1,092(62.6)	652(37.4)		
Duration of moderate-to-vigorous physical activity (MVPA)			245.985	<0.001
<30 min/day	14,711(75.7)	4,722(24.3)		
30–60 min/day	13,886(81.7)	3,119(18.3)		
>60 min/day	4,908(82.6)	1,036(17.4)		
Sugar-sweetened beverage intake			289.101	<0.001
≤2 times/week	11,467(81.1)	2,677(18.9)		
3–4 times/week	17,684(80.0)	4,419(20.0)		
≥5 times/week	4,354(71.0)	1781(29.0)		
Quartile of conicity index			47.544	<0.001
Quartile 1	8,436(79.5)	2,172(20.5)		
Quartile 2	8,488(80.5)	2061(19.5)		
Quartile 3	8,459(79.4)	2,190(20.6)		
Quartile 4	8,122(76.8)	2,454(23.2)		

Table 3 presents univariate analyses of the associations between SSB intake and quartiles of the CI with mental health symptoms and their various dimensions, stratified by the general population, males, and females. All χ^2^ test *p*-values were < 0.001. For both males and females, the higher the frequency of SSB consumption, the higher the prevalence of emotional, behavioral, and social adjustment difficulties, as well as overall mental health symptoms, with the prevalence being significantly highest among those consuming SSBs ≥5 times per week. Regarding the CI, the prevalence of symptoms across all dimensions continued to increase with higher CI quartiles among females; among males, only behavioral problems, social adjustment difficulties, and overall mental health symptoms increased with higher CI quartiles, while there was no between-group difference in emotional problems (*p* = 0.157). Stratification analysis revealed a stronger association between CI and emotional symptoms in females, suggesting that while the adverse effects of SSB on mental health symptoms do not differ by gender, the association between CI and mental health symptoms exhibits marked gender heterogeneity.

**Table 3:** Univariate comparison of SSB intake and CI with mental health symptoms among Chinese adolescents.

Classification	**Gender**	**χ2/P-value**	***N***	**Emotional problems**	**Behavioral problems**	**Social adjustment difficulties**	**Mental health symptoms**
Male	Sugar-sweetened beverage intake						
	≤2 times/week			1,622(26.3)	1,625(26.4)	1,062(17.2)	1,211(19.6)
	3–4 times/week			2,965(26.2)	3,049(26.9)	1912(16.9)	2,278(20.1)
	≥5 times/week			1,293(35.3)	1,270(34.7)	909(24.8)	1,031(28.2)
		χ^2^-value		124.66	95.662	123.964	122.015
		*P-*value		<0.001	<0.001	<0.001	<0.001
	Quartile of conicity index						
	Quartile 1			1,196(28.6)	1,220(29.1)	838(20.0)	874(20.9)
	Quartile 2			1,237(27.1)	1,245(27.3)	809(17.7)	898(19.7)
	Quartile 3			1,517(27.0)	1,520(27.1)	996(17.7)	1,177(21.0)
	Quartile 4			1930(28.4)	1959(28.8)	1,240(18.2)	1,571(23.1)
		χ^2^-value		5.21	8.418	10.359	21.269
		*P-*value		0.157	<0.001	0.016	<0.001
Female	Sugar-sweetened beverage intake						
	≤2 times/week			2006(25.1)	1841(23.1)	1,208(15.1)	1,466(18.4)
	3–4 times/week			2,922(27.1)	2,662(24.7)	1,698(15.8)	2,141(19.9)
	≥5 times/week			937(37.9)	890(36.0)	630(25.5)	750(30.3)
		χ^2^-value		156.232	171.781	157.966	171.095
		*P-*value		<0.001	<0.001	<0.001	<0.001
	Quartile of conicity index						
	Quartile 1			1754(27.3)	1,614(25.1)	1,087(16.9)	1,298(20.2)
	Quartile 2			1,577(26.3)	1,421(23.7)	928(15.5)	1,163(19.4)
	Quartile 3			1,361(27.0)	1,259(25.0)	816(16.2)	1,013(20.1)
	Quartile 4			1,173(31)	1,099(29.1)	705(18.6)	883(23.4)
		χ^2^-value		27.981	36.213	17.608	23.870
		*P-*value		<0.001	<0.001	0.001	<0.001
Total	Sugar-sweetened beverage intake						
	≤2 times/week			3,628(25.7)	3,466(24.5)	2,270(16.0)	2,677(18.9)
	3–4 times/week			5,887(26.6)	5,711(25.8)	3,610(16.3)	4,419(20.0)
	≥5 times/week			2,230(36.3)	2,160(35.2)	1,539(25.1)	1781(29.0)
		χ^2^-value		271.267	269.736	285.929	289.101
		*P-*value		<0.001	<0.001	<0.001	<0.001
	Quartile of conicity index						
	Quartile 1			2,950(27.8)	2,834(26.7)	1925(18.1)	2,172(20.5)
	Quartile 2			2,814(26.7)	2,666(25.3)	1737(16.5)	2061(19.5)
	Quartile 3			2,878(27.0)	2,779(26.1)	1812(17.0)	2,190(20.6)
	Quartile 4			3,103(29.3)	3,058(28.9)	1945(18.4)	2,454(23.2)
		χ^2^-value		22.201	39.369	18.421	47.544
		*P-*value		<0.001	<0.001	<0.001	<0.001

Table 4 presents the results of stratified logistic regression analysis, in which three stepwise adjustment models were constructed for male students, female students, and the overall population, respectively, to examine the independent associations between SSB intake, the CI, and mental health symptoms. Model 1 was the unadjusted model; Model 2 was adjusted for age; and Model 3 was further adjusted for mode of commuting to school, breakfast frequency, and moderate-to-vigorous physical activity.

**Table 4:** Logistic regression analysis of SSB intake and CI among Chinese adolescents and their mental health symptoms.

Gender/Variable	Group	Mental health symptoms					
		Model 1		Model 2		Model 3	
		OR (95% CI)	*P-*value	OR (95% CI)	*P-*value	OR (95% CI)	*P-*value
Male							
Sugar-sweetened beverage intake	≤2 times/week	1.00		1.00		1.00	
	3–4 times/week	1.03(0.95 ~ 1.11)	0.458	1.03(0.95 ~ 1.11)	0.472	1.04(0.96 ~ 1.12)	0.376
	≥5 times/week	1.60(1.46 ~ 1.76)	<0.001	1.60(1.45 ~ 1.76)	<0.001	1.53(1.38 ~ 1.68)	<0.001
Quartile of conicity index	Quartile 1	1.00		1.00		1.00	
	Quartile 2	0.93(0.84 ~ 1.03)	0.163	0.92(0.83 ~ 1.03)	0.14	0.95(0.86 ~ 1.06)	0.341
	Quartile 3	1.01(0.91 ~ 1.11)	0.910	1.00(0.91 ~ 1.11)	0.983	1.04(0.94 ~ 1.15)	0.404
	Quartile 4	1.14(1.04 ~ 1.25)	0.006	1.14(1.04 ~ 1.25)	0.007	1.17(1.06 ~ 1.28)	0.001
Female							
Sugar-sweetened beverage intake	≤2 times/week	1.00		1.00		1.00	
	3–4 times/week	1.10(1.02 ~ 1.19)	0.01	1.10(1.02 ~ 1.19)	0.01	1.08(1.00 ~ 1.16)	0.054
	≥5 times/week	1.93(1.75 ~ 2.14)	<0.001	1.94(1.75 ~ 2.14)	<0.001	1.83(1.65 ~ 2.03)	<0.001
Quartile of conicity index	Quartile 1	1.00		1.00		1.00	
	Quartile 2	0.95(0.87 ~ 1.04)	0.27	0.95(0.87 ~ 1.04)	0.27	0.95(0.87 ~ 1.04)	0.237
	Quartile 3	0.99(0.91 ~ 1.09)	0.894	0.99(0.91 ~ 1.09)	0.896	0.99(0.91 ~ 1.09)	0.905
	Quartile 4	1.20(1.09 ~ 1.33)	<0.001	1.20(1.09 ~ 1.33)	<0.001	1.18(1.07 ~ 1.30)	0.001
Total							
Sugar-sweetened beverage intake	≤2 times/week	1.00		1.00		1.00	
	3–4 times/week	1.07(1.02 ~ 1.13)	0.013	1.07(1.01 ~ 1.13)	0.014	1.06(1.00 ~ 1.12)	0.037
	≥5 times/week	1.75(1.64 ~ 1.88)	<0.001	1.75(1.63 ~ 1.87)	<0.001	1.66(1.54 ~ 1.78)	<0.001
Quartile of conicity index	Quartile 1	1.00		1.00		1.00	
	Quartile 2	0.94(0.88 ~ 1.01)	0.088	0.94(0.88 ~ 1.01)	0.076	0.95(0.89 ~ 1.02)	0.129
	Quartile 3	1.01(0.94 ~ 1.08)	0.871	1.00(0.94 ~ 1.07)	0.968	1.02(0.95 ~ 1.09)	0.566
	Quartile 4	1.17(1.10 ~ 1.25)	<0.001	1.17(1.09 ~ 1.25)	<0.001	1.17(1.09 ~ 1.25)	<0.001

Model 1 is crude; Model 2 adjusts for age based on Model 1; Model 3 adjusts for “Commuting to and from school,” “Frequency of breakfast,” and “Duration of moderate-to-vigorous physical activity” based on Model 2.

Regarding SSB consumption, using consumption of ≤2 times per week as the reference, high-frequency consumption (≥5 times per week) was significantly associated with an increased risk of mental health symptoms in all models for the overall population, males, and females (*p <* 0.001); in the fully adjusted model for the overall population, the OR was 1.66; among females, consumption 3–4 times per week was associated with an increased risk in the crude model; after adjusting for all confounders, the association weakened to borderline significance (*p* = 0.054); among males, moderate-frequency consumption showed no statistically significant difference.

For CI, using the lowest quartile (Q1) as the reference, only the highest quartile (Q4) was an independent risk factor in all models; the adjusted OR for the overall population was 1.17, with consistent trends observed among both male and female adolescents; Q2 and Q3 were not significantly associated with mental health symptoms.

Overall, the results suggest that high-frequency SSB intake and high CI are independently associated with mental health symptoms among adolescents. A dose–response relationship exists for SSB intake, and the effect varies by gender, with females being more sensitive to moderate-frequency SSB intake.

Table 5 presents the results of an ordered logistic regression analysis of the combined effect of the two factors, adjusted for all confounding factors, with “SSB ≤ 2 times/week + CI Q1” as the reference group. In the overall population, SSB ≥ 5 times/week combined with any CI quartile significantly increased the risk of mental health symptoms, with the highest risk observed when high SSB was combined with the highest CI quartile (OR = 2.12, *p <* 0.001). Gender-specific analysis revealed marked heterogeneity: among males, risk was elevated only in groups with high-frequency SSB consumption, and no gradient of combined effects was observed across CI quartiles. Among females, the combined effect was more pronounced, with an OR of 3.41 for the combination of high-frequency SSB consumption and the highest CI quartile. For combinations of low- and moderate-frequency SSB consumption with various CI groups, most groups showed no significant increase in risk; only the combination of moderate-frequency SSB consumption and high CI showed a slight increase in risk in the overall population. These findings indicate a positive combined association between high SSB intake and high CI, which appears more pronounced among female adolescents. Formal interaction tests (RERI and the multiplicative interaction term) were non-significant in the overall sample; the apparent additive pattern should therefore be interpreted as exploratory. The trends in OR values are shown in Figure 1.

**Table 5:** Analysis of the combined effects of SSB intake and CI on mental health symptoms among Chinese adolescents.

Gender	Classification of interaction		Mental health symptoms	
	Sugar-sweetened beverage intake	Quartile of Conicity Index	OR (95% CI)	*P*-value
Male	≤2 times/week	Quartile 1	1.00	
		Quartile 2	0.83(0.68 ~ 1.01)	0.068
		Quartile 3	1.01(0.84 ~ 1.22)	0.876
		Quartile 4	1.08(0.91 ~ 1.30)	0.373
	3–4 times/week	Quartile 1	0.96(0.81 ~ 1.15)	0.668
		Quartile 2	0.94(0.79 ~ 1.12)	0.505
		Quartile 3	0.98(0.83 ~ 1.16)	0.809
		Quartile 4	1.15(0.98 ~ 1.35)	0.082
	≥5 times/week	Quartile 1	1.61(1.29 ~ 1.99)	<0.001
		Quartile 2	1.49(1.21 ~ 1.85)	<0.001
		Quartile 3	1.55(1.27 ~ 1.89)	<0.001
		Quartile 4	1.68(1.40 ~ 2.03)	<0.001
Female	≤2 times/week	Quartile 1	1.00	
		Quartile 2	0.97(0.84 ~ 1.13)	0.688
		Quartile 3	0.90(0.77 ~ 1.06)	0.212
		Quartile 4	1.14(0.96 ~ 1.34)	0.132
	3–4 times/week	Quartile 1	1.09(0.95 ~ 1.25)	0.203
		Quartile 2	1.03(0.89 ~ 1.18)	0.708
		Quartile 3	1.12(0.97 ~ 1.29)	0.114
		Quartile 4	1.16(0.99 ~ 1.35)	0.059
	≥5 times/week	Quartile 1	1.58(1.31 ~ 1.91)	<0.001
		Quartile 2	1.58(1.3 ~ 1.94)	<0.001
		Quartile 3	1.90(1.54 ~ 2.34)	<0.001
		Quartile 4	3.41(2.75 ~ 4.23)	<0.001
Total	≤2 times/week	Quartile 1	1.00	
		Quartile 2	0.92(0.81 ~ 1.03)	0.16
		Quartile 3	0.97(0.86 ~ 1.09)	0.574
		Quartile 4	1.13(1.00 ~ 1.27)	0.048
	3–4 times/week	Quartile 1	1.04(0.94 ~ 1.16)	0.45
		Quartile 2	1.00(0.89 ~ 1.11)	0.945
		Quartile 3	1.06(0.95 ~ 1.18)	0.293
		Quartile 4	1.18(1.07 ~ 1.31)	0.002
	≥5 times/week	Quartile 1	1.61(1.40 ~ 1.85)	<0.001
		Quartile 2	1.56(1.35 ~ 1.80)	<0.001
		Quartile 3	1.71(1.48 ~ 1.96)	<0.001
		Quartile 4	2.12(1.86 ~ 2.42)	<0.001

In the ordered logistic regression model, the variables age, commuting to and from school, Frequency of breakfast, and Duration of moderate-to-vigorous physical activity were adjusted for.

**Figure 1:**
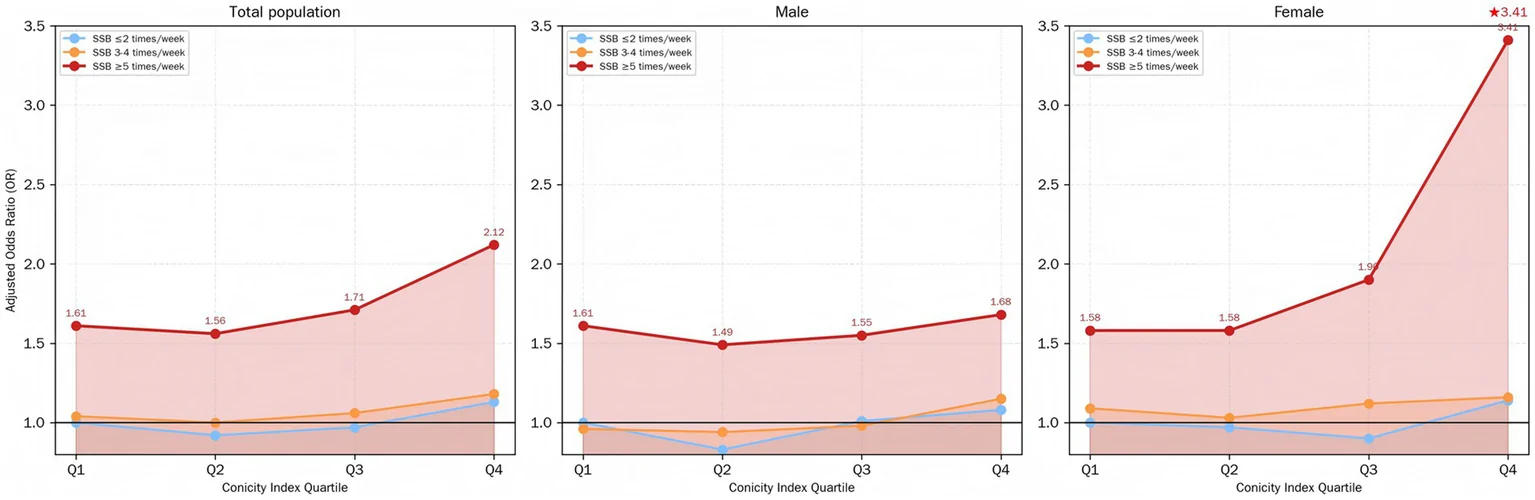
Trend chart of OR values in the analysis of the combined effects of SSB intake and CI on mental health symptoms among Chinese adolescents.

## 4 Discussion

This study found that the overall prevalence of mental health symptoms among Chinese adolescents aged 12–17 was 20.9%, with boys (21.4%) showing a slightly higher rate than girls (20.5%). These findings are generally consistent with data from several previous nationwide surveys. A systematic review covering mainland China indicated that the overall prevalence of depressive symptoms among middle school students was approximately 26.17%(Zhou et al., 2024). Longitudinal studies indicate that the prevalence of depressive symptoms followed an inverted U-shaped trend from 2019 to 2023, rising in 2020–2021 and then declining in 2022–2023 (Zhu et al., 2025). Globally, data from the World Health Organization indicate that approximately 10 to 20% of adolescents have experienced at least one form of psychosocial health issue. In 2021, the age-standardized prevalence of depression among adolescents and young people aged 10–24 worldwide was approximately 14,764.94 per 100,000 (Wang Z. et al., 2025). Although our observed prevalence (20.9%) appears to be at the upper end of the WHO range (10–20%) and is broadly consistent with previous nationwide surveys in China, the cross-study comparability is constrained by differences in sampling frames, screening instruments, and case definitions; therefore the detection rate should be interpreted with caution rather than as a direct ranking against global data. Regarding gender differences, the detection rate of mental health symptoms was higher among boys than among girls. This is consistent with the results of some nationwide surveys that used the BIOPHY instrument, but differs from the findings in most studies focusing on depression and anxiety as core outcomes, where women typically score higher than men. This discrepancy may be related to differences in the conceptual content of the assessment tools. The BIOPHY includes dimensions of conduct problems and social adjustment difficulties, and boys typically score higher than girls on externalizing behavior problems, thereby raising the overall detection rate among boys.

This study found a significant independent association between SSB intake and mental health symptoms in adolescents, with a clear dose–response relationship. In the overall population, individuals who consumed SSBs ≥5 times per week had a 66% higher risk of mental health symptoms compared to those who consumed them ≤2 times per week (OR = 1.66); After stratification by gender, the risk associated with high-frequency consumption was higher among girls (OR = 1.83) than among boys (OR = 1.53). Furthermore, among girls, moderate-frequency consumption (3–4 times per week) remained at the borderline level of significance (*p* = 0.054) even after adjusting for all confounding factors. In contrast, among boys, moderate-frequency consumption was consistently not statistically significant. This finding is highly consistent with the international literature. Khaled et al. found that high SSB intake was significantly associated with a 34% increased risk of anxiety disorders in adolescents (OR = 1.34); longitudinal studies further confirmed that this association persisted during follow-up periods of more than 1 year (Khaled et al., 2026). A nationwide survey in South Korea found that adolescents who consumed SSBs 5–6 times per week had significantly higher odds of perceived stress (AOR = 1.35), depressive symptoms (AOR = 1.32), suicidal ideation (AOR = 1.23), and feelings of loneliness (AOR = 1.44) (Lee et al., 2025). From a physiological perspective, SSBs may affect mental health through multiple mechanisms. High sugar intake causes blood glucose levels to rise rapidly and then drop sharply, leading to mood swings, irritability, and fatigue, while also disrupting the normal synthesis and release of neurotransmitters such as dopamine (Aguilera et al., 2000). High fructose intake can induce neuroinflammatory responses in brain regions involved in emotion regulation, such as the hippocampus and prefrontal cortex, thereby impairing neuronal plasticity and synaptic function (Fierros-Campuzano et al., 2022). A high-sugar diet can disrupt the function of the hypothalamic–pituitary–adrenal axis, leading to persistently elevated cortisol levels and exacerbating anxiety and depression-like behaviors (Smith et al., 2023). In addition, unabsorbed sugars alter the composition of the gut microbiota, increase intestinal permeability, and trigger systemic chronic inflammation; inflammatory factors then cross the blood–brain barrier and affect central nervous system function (Proctor et al., 2017). Among the gender differences observed in this study, women were more sensitive to moderate-frequency SSB consumption. This finding may be related to the timing of maturation in brain regions involved in emotional regulation—such as the prefrontal cortex and the amygdala—as well as the modulatory effects of estrogen on inflammatory responses (Della Corte et al., 2020). In addition, adolescent girls are more sensitive to changes in body image and weight during puberty, and weight gain resulting from SSB consumption may further exacerbate their psychological burden through psychosocial mechanisms (Baceviciene et al., 2023). It is worth noting that most of the evidence supporting the aforementioned mechanisms comes from animal experiments and cross-sectional studies; the complete biological pathways by which SSBs affect mental health still need to be elucidated through longitudinal studies and neuroimaging research.

This study found that the highest quartile (Q4) of central obesity (CI) was an independent risk factor for mental health symptoms, with an adjusted odds ratio (OR) of 1.17 in the overall population. At the same time, Q2 and Q3 were not significantly associated with mental health symptoms. This pattern is more consistent with a threshold-type relationship rather than a linear dose–response gradient: Q2 and Q3 showed no significant association, while only the top quartile (Q4) was independently associated with mental health symptoms, suggesting that the adverse psychological correlates of central adiposity may only emerge once visceral fat accumulation reaches a clinically meaningful level. We interpret this finding as biologically plausible, because visceral adipose tissue becomes metabolically and immunologically dysregulated above a certain mass, leading to a marked increase in systemic low-grade inflammation and cortisol reactivity—both of which have been linked to depressive and anxiety symptoms in adolescents. However, the cross-sectional nature of the present analysis precludes any inference about causality, and the relatively modest effect size of the top CI quartile (adjusted OR ≈ 1.17) warrants cautious interpretation: this corresponds to a 17% relative increase in odds at the population level and should not be translated into individual-level clinical decisions. CI was first proposed by Mueller et al. (1996). Compared with BMI, CI more accurately reflects the degree of visceral fat accumulation and the characteristics of body fat distribution by incorporating waist circumference, height, and weight (Mueller et al., 1996). Previous studies have shown that CI is closely associated with various metabolic disorders, including adolescent metabolic syndrome, dyslipidemia, and hepatic steatosis. In the field of mental health, a study of 42,472 Chinese adolescents aged 12 to 18 found that BMI, WC, WHtR, and BRI were all positively correlated with mental health symptoms, and that these new obesity indicators may be more accurate than traditional BMI in identifying mental health issues. Another study of Chinese adolescents found that waist circumference partially mediates the relationship between screen time and mental health; that is, excessive screen time may indirectly harm mental health by increasing waist circumference (Wang et al., 2025). The potential mechanisms by which central obesity affects mental health involve multiple factors. First, visceral adipose tissue is highly metabolically active. It secretes large amounts of pro-inflammatory cytokines, including interleukin-6 (IL-6), tumor necrosis factor-α (TNF-α), and C-reactive protein (CRP), thereby inducing a state of chronic, low-grade systemic inflammation. These inflammatory factors can influence neurotransmitter metabolism by crossing the blood–brain barrier, particularly by reducing serotonin synthesis via the tryptophan-kynurenine pathway, thereby directly contributing to the pathophysiological processes underlying depression and anxiety (Lama et al., 2022). Second, central obesity is often accompanied by insulin resistance and leptin resistance; these hormonal imbalances can interfere with the normal functioning of the hypothalamic centers responsible for energy metabolism and emotional regulation (Tian et al., 2025). Finally, psychosocial factors such as obesity-related social stigma, body dissatisfaction, peer rejection, and low self-esteem may exacerbate psychological distress through chronic psychosocial stress. This phenomenon is particularly pronounced during adolescence, a time when adolescents are highly sensitive to their appearance (Ozbardakci and Lydecker, 2023). The gender-stratified analysis in this study revealed significant effect moderation. The association between CI and emotional problems in girls increased progressively across quartiles and was statistically significant (*p <* 0.001), whereas no significant differences were observed in emotional problems among boys across CI groups (*p* = 0.157); only behavioral problems, social adjustment difficulties, and overall mental health symptoms increased with rising CI. This gender heterogeneity may be because adolescent girls are more socioculturally sensitive to changes in body fat distribution than boys, and the body shape changes associated with central obesity are more likely to trigger body dissatisfaction and emotional distress in girls (Moize et al., 2025). In addition, estrogen may influence emotional regulation by modulating inflammatory responses and neuroplasticity, making women more sensitive to the neurobehavioral effects of obesity-related metabolic disorders (Mina et al., 2014). These findings suggest that, in mental health interventions for adolescents, gender-specific strategies for body fat management should be developed, with greater emphasis placed on the psychosocial impacts of central obesity and emotional support for girls.

Analysis of combined effects revealed that SSB intake and CI show a combined association with mental health symptoms. In the overall population, the risk was highest among those with high-frequency SSB intake and the highest CI quartile (OR = 2.12), which was significantly greater than the sum of the ORs for the two factors acting independently, consistent with an additive pattern between the two exposures (RERI not significant). A gender-stratified analysis revealed that this combined effect appeared more pronounced among women, with an OR of 3.41 for frequent SSB consumption combined with the highest CI quartile, compared with 1.68 among men. The gender-stratified analyses should be considered exploratory subgroup analyses rather than evidence of a formally tested statistical interaction, because the multiplicative interaction term for sex was not significant in our revised model (*p* > 0.05). This finding suggests that SSB consumption and central obesity may produce an additive effect through common biological pathways, such as inflammatory pathways; SSB-induced neuroinflammation and visceral fat-related systemic inflammation may jointly act on the brain’s emotional regulation centers (Laugero and Keim, 2023). The combined effect, which appears more pronounced among women, may be related to estrogen’s regulatory role in the inflammatory response and to women’s greater psychosocial sensitivity to changes in body fat distribution (Pei et al., 2017).

This study employed a large-scale, nationwide, multistage, stratified, cluster-randomized sampling design with a sample size of 42,382 participants, covering China’s five major geographic regions and demonstrating good national representativeness. However, this study also has some limitations. First, the cross-sectional study design does not allow for inferring a causal relationship between SSB intake, CI, and mental health symptoms; the direction of the association requires further validation through longitudinal studies. Future prospective cohort studies and randomized controlled trials are needed to validate further the causal effects of SSB intake and CI on mental health symptoms, as well as their underlying biological mechanisms, and to explore potential intervention strategies targeting the reduction of neuroinflammation. Second, both the SSB intake frequency and the mental health symptoms were assessed using self-report questionnaires, which are vulnerable to recall bias, social desirability bias, and reporting bias; even with validated instruments (e.g., HBSC, BIOPHY), under-reporting of SSB consumption and over- or under-reporting of symptom severity cannot be ruled out and may have attenuated or inflated the observed associations. In addition, the SSB exposure was operationalized as intake frequency rather than quantity, and the highest two categories (5–6 times/week and ≥7 times/week) were collapsed; this grouping is likely to obscure any dose–response pattern within the upper tail of the SSB distribution and may have introduced exposure misclassification. Third, residual confounding cannot be excluded. Although we adjusted for a more comprehensive set of covariates in the revised analysis (including sleep duration, parental education, academic pressure, smoking, and alcohol use), other potential confounders—such as overall dietary quality, family history of mental illness, and genetic susceptibility— were not measured and may bias the observed effect estimates. Fourth, the exclusion of adolescents with self-reported prior psychiatric diagnoses may have lowered the prevalence of mental health symptoms and limited the external validity of the findings to a healthier subset of the adolescent population. Fifth, the adjusted odds ratio for the highest CI quartile (OR ≈ 1.17) was relatively modest; given the cross-sectional design, this effect size should be interpreted as a population-level association rather than a clinically actionable individual-level effect, and its public-health significance remains to be confirmed in longitudinal and intervention studies. Sixth, while CI is a good indicator of central obesity, as an indirect anthropometric measure, it cannot precisely distinguish between visceral and subcutaneous fat; future studies may incorporate imaging techniques to verify these findings.

## 5 Conclusion

The prevalence of mental health symptoms among Chinese adolescents is relatively high. Both SSB intake and CI are independent risk factors for mental health symptoms, and there is a significant synergistic effect between the two, which is particularly pronounced among females. It is recommended that schools restrict the availability of SSBs on campus and promote healthy beverage alternatives. Additionally, early screening for central obesity and weight management among adolescents should be strengthened, and mental health promotion should be incorporated into routine school health programs. Given gender differences, tailored intervention strategies should be developed, with a greater emphasis on combined interventions targeting SSB intake control and body fat management for female adolescents.

## Data Availability

The raw data supporting the conclusions of this article will be made available by the authors, without undue reservation.
